# Systematic pseudopotentials from reference eigenvalue sets for DFT calculations: Pseudopotential files

**DOI:** 10.1016/j.dib.2014.12.005

**Published:** 2015-01-13

**Authors:** Pablo Rivero, Víctor Manuel García-Suárez, David Pereñiguez, Kainen Utt, Yurong Yang, Laurent Bellaiche, Kyungwha Park, Jaime Ferrer, Salvador Barraza-Lopez

**Affiliations:** a Department of Physics, University of Arkansas, Fayetteville, AR 72701, USA; b Departamento de Física, Universidad de Oviedo and Centro de Investigación en Nanotecnología, Oviedo, Spain; c Physics Department, Virginia Polytechnic Institute and State University, Blacksburg, VA 24061, USA

## Abstract

We present in this article a pseudopotential (PP) database for DFT calculations in the context of the SIESTA code [1–3]. Comprehensive optimized PPs in two formats (psf files and input files for *ATM* program) are provided for 20 chemical elements for LDA and GGA exchange-correlation potentials. Our data represents a validated database of PPs for SIESTA DFT calculations. Extensive transferability tests guarantee the usefulness of these PPs.

**Table:** **Specificationstable**

Subject area	*Physics, chemistry*
More specific subject area	*Computational condensed matter*
Type of data	*SIESTA pseudopotential database:1. Input files for the atom program 2. psf files for direct use in the SIESTA code. 3. Band structures for these materials for which the improvement in the description is small.*
How data was acquired	*By a computational method developed to optimize SIESTA pseudopotentials*
Data format	*Raw text files*
Experimental factors	–
Experimental features	*The pseudopotentials have been generated by an iterative method that match eigenvalues and lattice parameters to reference VASP eigenvalues*
Data source location	*Fayetteville, AR, USA*
Data accessibility	*Data are available here with this article*

## Value of the data

• The electronic dispersion and structural properties for Ge, Pd, Pt, Au, Ag, and Ta have a better agreement with respect to all-electron results now.
• We provide the expected metallic behavior of Sn in the bulk, which comes out semiconducting when using available pseudopotentials.
• We create a validated pseudopotential for LDA-tungsten.
• We create the first Bi pseudopotential for SIESTA that reproduces well-known electron and hole pockets at the L and T points.

## 1 Explored bulk materials

We obtained pseudopotentials (PPs) for DFT calculations within the context of the SIESTA code [1-3]. We extend the ongoing verification effort of Ref. [4] and corroborate that, out of 20 explored elements, the PPs available for C (atomic number 6), Al (13), Si (14), V (23), Cr (24), Cu (29), Se (34), Nb (41), and Te (52) perform well for both LDA and GGA (PBE) exchange-correlation potentials when compared to VASP [5] results. This excellent performance occurs regardless of the specific basis set employed as long as the basis set is of DZP or larger size. At the same time, the PPs for Ge (32), Pd (46), Ag (47), Sn (50), Sb (51), Ta (73), Pt (78), Au (79), and Bi (83) in Ref. [4] can be improved and we demonstrate a viable route towards solving some of their issues regarding lattice constants and electronic structure.

## 2 The algorithm

We generate PPs on the fly by matching eigenvalues and lattice parameters versus VASP calculations with PAW PPs [5] which: (a) reproduce all-electron results reasonably well, albeit at a cheaper computational price, and (b) are available for all chemical elements, for many exchange-correlation potentials, and for many valence electronic configurations.

Our algorithm, described in Ref. [7], works as follows: one runs VASP first to obtain an EIGENVAL file on the equilibrium structure. Upon execution, our algorithm first calls *ATM* program to produce a SIESTA pseudopotential (psf file) from the INP file. With the psf file just created the algorithm calls SIESTA in order to generate an eigenvalue output file (EIG) on a dense *k*-point mesh using the psf and basis.fdf files. The algorithm records the absolute differences among the eigenvalues on the EIG file and those stored on a VASP׳s EVALUES file, over nb bands around the Fermi level.

Based upon the Simplex algorithm [6] for the optimization in multidimensional spaces, the code establishes a new set of input parameters to continue to optimization cycle, and *ATM* and SIESTA continue to be called until the Simplex search reaches a point of no further improvement of the SIESTA eigenvalue sets. At that point the code stops, and we retrieve the psf file that leads to the most optimized pseudopotential. Further checks were carried out, as indicated in the main manuscript.

## References

[1] Soler J., Artacho E., Gale J., García A., Junquera J., Ordejón P., Sánchez-Portal D. (2002). J. *Phys.: Condens. Matter*.

[2] Artacho E., Anglada E., Dieguez O., Gale J., García A., Junquera J., Martin R., Ordejón P., Pruneda J., Sánchez-Portal D. (2008). J. Phys.: Condens. Matter.

[3] Artacho E., Sánchez-Portal D., Ordejón P., García A., Soler J. (1999). Phys. Stat. Sol. B.

[4] 〈http://departments.icmab.es/leem/siesta/Databases/index.html〉.

[5] Kresse G., Joubert J. (1999). Phys. Rev. B.

[6] Press W., Flannery B., Teukolsky S., Vetterling W. (1989). Numerical Recipes.

[7] Rivero P., Garcia-Suarez V.M., Pereñiguez D., Utt K., Yang Y., Bellaiche L., Park K., Ferrer J., Barraza-Lopez. S. (2015). Comp. Mater. Sci..

