# Supplementary material for: Systematic pseudopotentials from reference eigenvalue sets for DFT calculations: Pseudopotential files
Source: Data Brief. 2015 Jan 13;3:21–3. doi: 10.1016/j.dib.2014.12.005 (PMC4509927; doi:10.1016/j.dib.2014.12.005)
Supplement: Supplementary file 1 — Supplementary data [file mmc1.zip › AmoebaCMP_4_SI.pdf]

Systematic pseudopotentials from reference eigenvalue sets for DFT calculations
Additional information

Pablo Rivero[a], Víctor Manuel García-Suárez[b], David Pereñiguez[a], Kainen Utt[a], Yurong Yang[a], Laurent Bellaiche[a], Kyungwha Park[c], Jaime Ferrer[b], Salvador Barraza-Lopez[a]

[a]*Department of Physics. University of Arkansas. Fayetteville AR, 72701. USA*
[b]*Departamento de Física, Universidad de Oviedo and Centro de Investigación en Nanotecnología. Oviedo, SPAIN*
[c]*Physics Department. Virginia Polytechnic Institute and State University. Blacksburg VA, 24061. USA*

Keywords: A. Pseudopotentials, B. Numerical atomic orbitals, C. Density-functional theory.

We display in the following pages those elements for which we are unable to improve the electronic dispersion in a significant manner: C, Al, Si, V, Cr, Cu, Se, Nb, and Te. Please refer to Table 2 in the main text to assess the overall improvement by means of the ratio Y_o/Y_i.

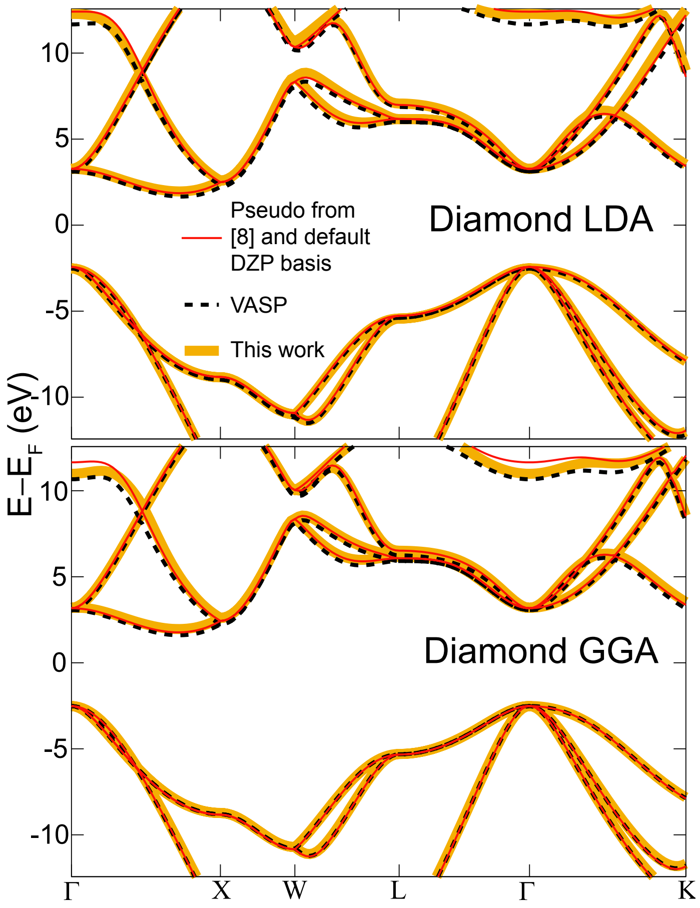

Figure 1: C (Z = 6)

URL: sbarraza@uark.edu (Salvador Barraza-Lopez)

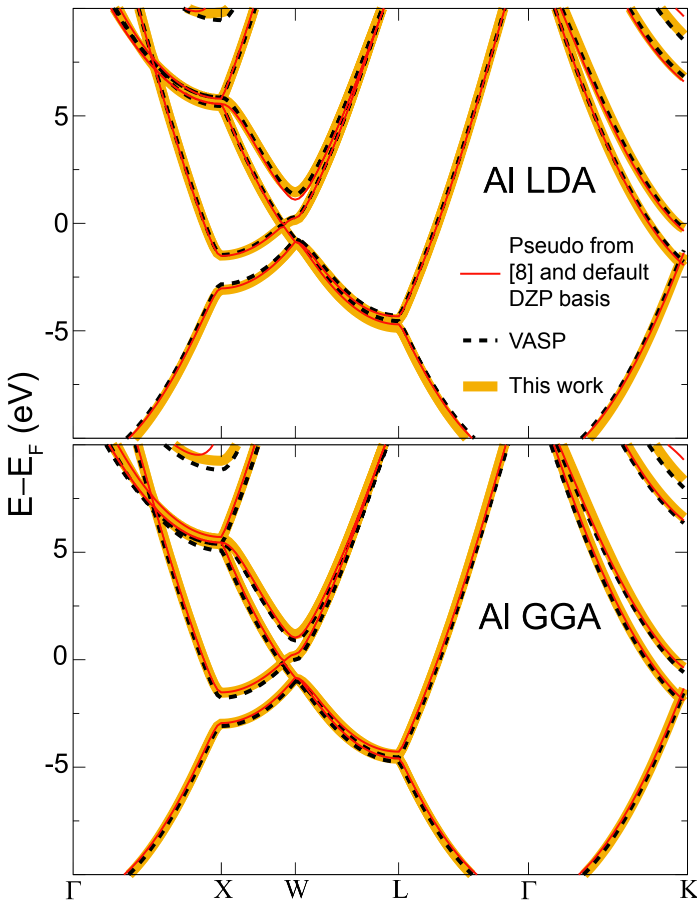

Figure 2: Al (Z = 13)

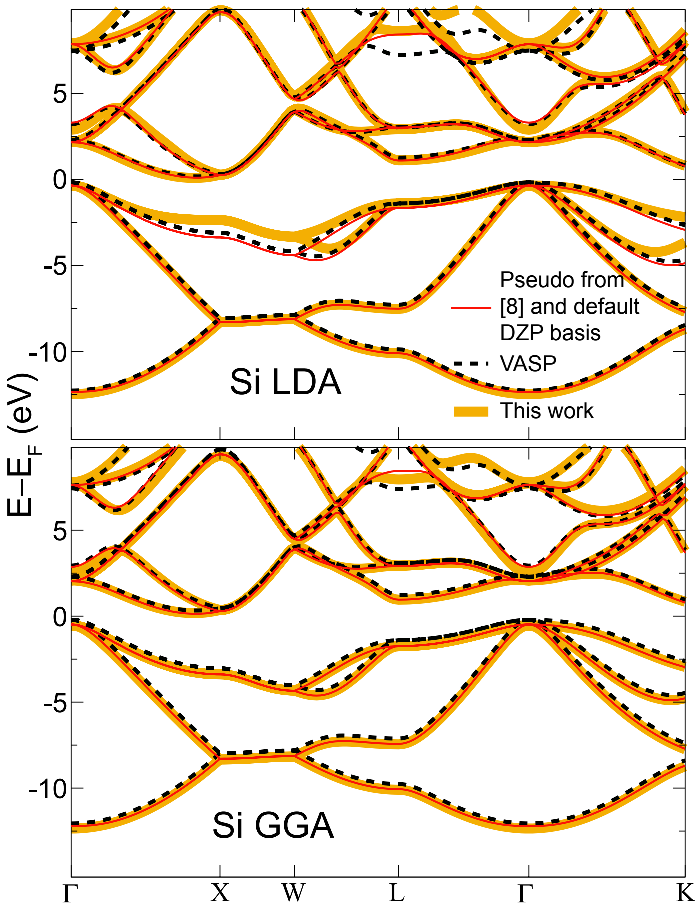

Figure 3: Si (Z = 14).

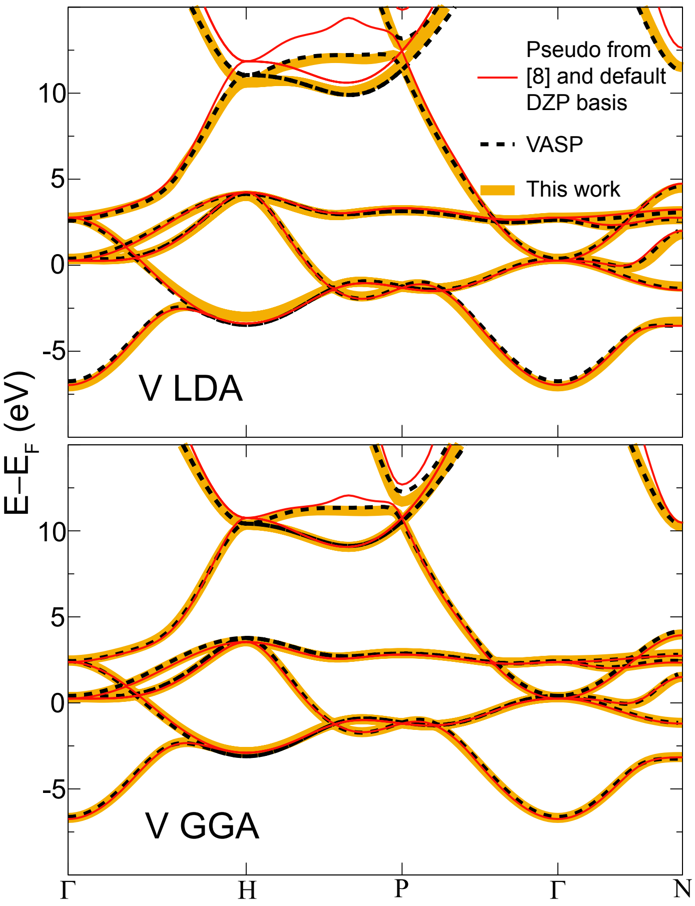

Figure 4: V (Z = 23).

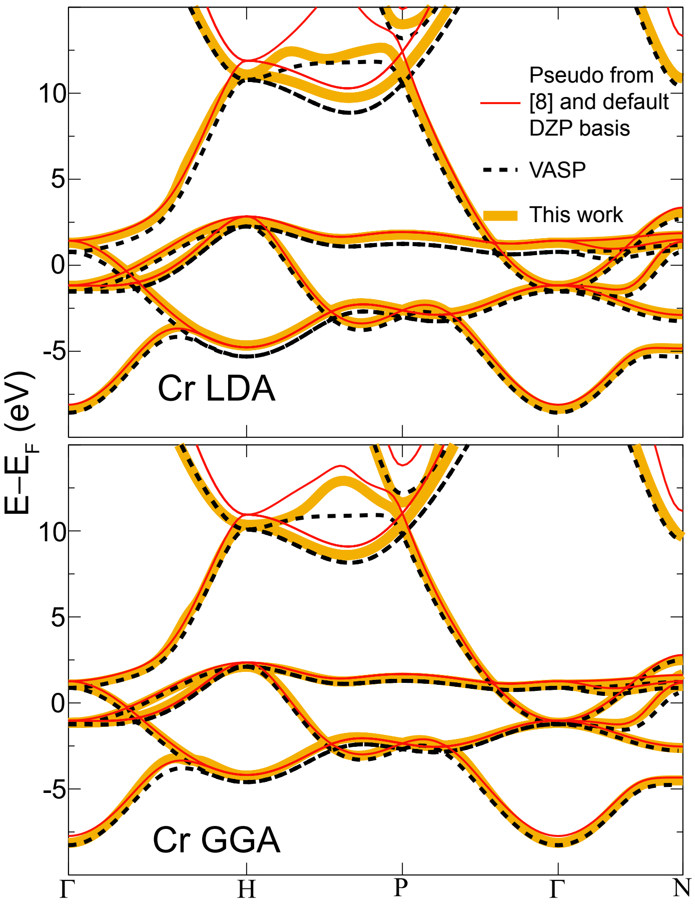

Figure 5: Cr (Z = 24).

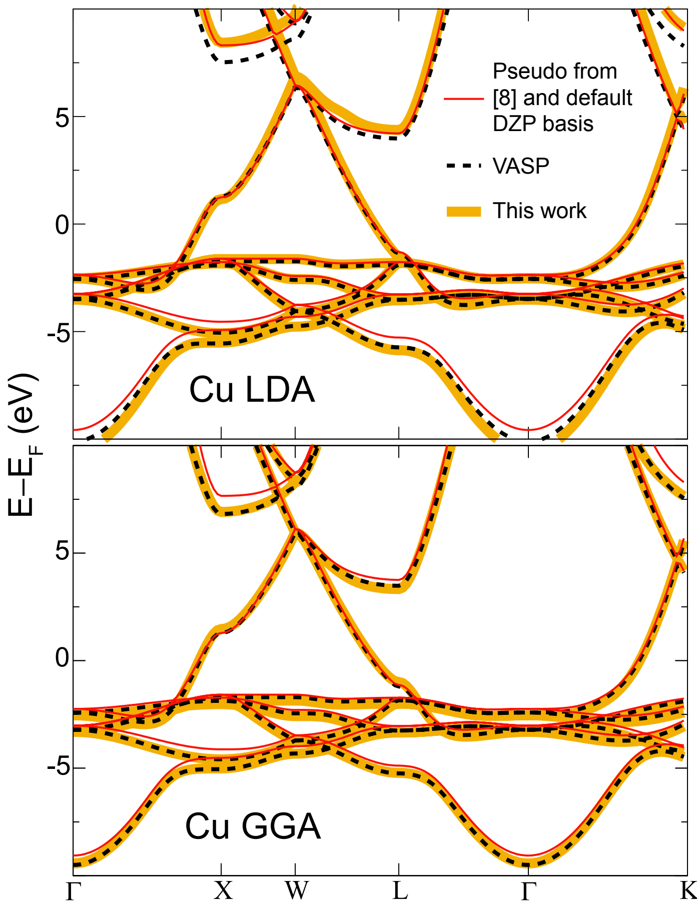

Figure 6: Cu (Z = 29).

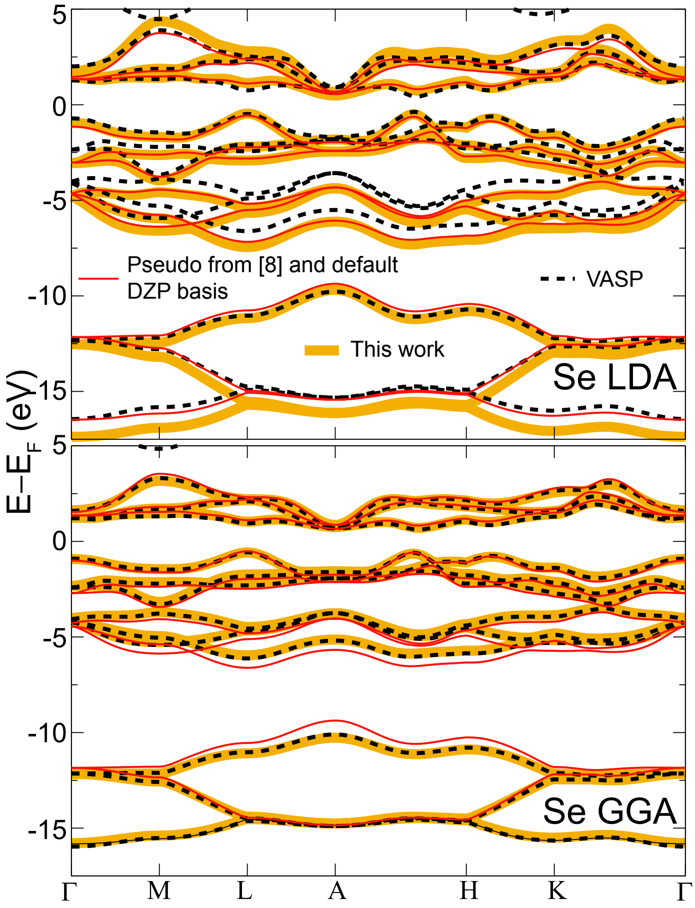

Figure 7: Se (Z = 34).

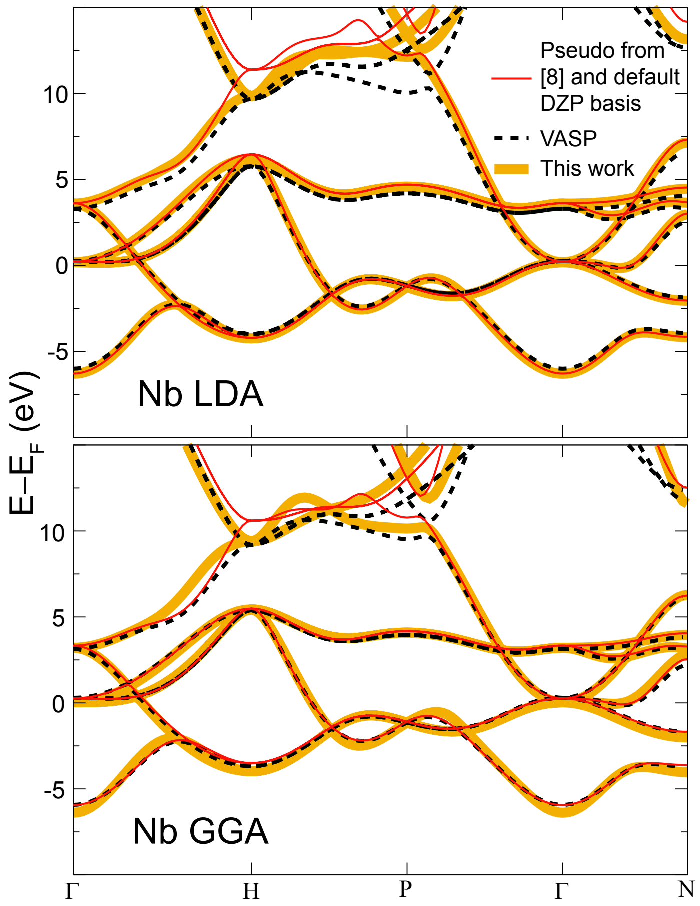

Figure 8: Nb (Z = 41).

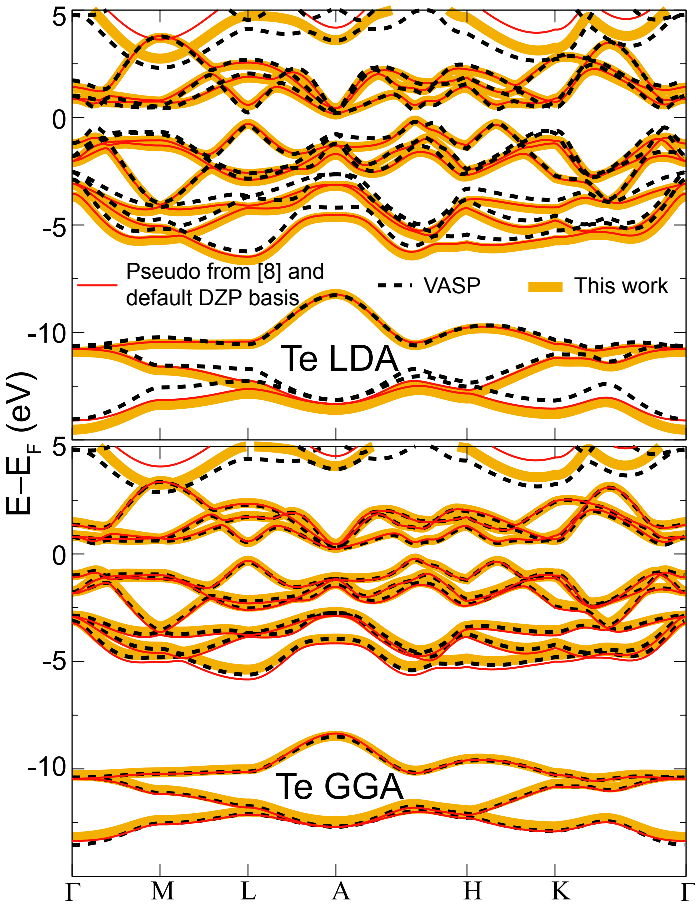

Figure 9: Te (Z = 52).
